# The complete mitochondrial genome sequence of the hydrothermal vent galatheid crab *Shinkaia crosnieri *(Crustacea: Decapoda: Anomura): A novel arrangement and incomplete tRNA suite

**DOI:** 10.1186/1471-2164-9-257

**Published:** 2008-05-30

**Authors:** Jin-Shu Yang, Wei-Jun Yang

**Affiliations:** 1 Institute of Cell Biology and Genetics, College of Life Sciences, Zijingang Campus, Zhejiang University, Hangzhou, Zhejiang 310058, PR China; 2 State Conservation Center for Gene Resources of Wildlife and the Key Laboratory of Conservation Genetics and Reproductive Biology for Wild Animals of the Ministry of Education, Hangzhou, Zhejiang 310058, PR China

## Abstract

**Background:**

Metazoan mitochondrial genomes usually consist of the same 37 genes. Such genes contain useful information for phylogenetic analyses and evolution modelling. Although complete mitochondrial genomes have been determined for over 1,000 animals to date, hydrothermal vent species have, thus far, remained excluded due to the scarcity of collected specimens.

**Results:**

The mitochondrial genome of the hydrothermal vent galatheid crab *Shinkaia crosnieri *is 15,182 bp in length, and is composed of 13 protein-coding genes, two ribosomal RNA genes and only 18 transfer RNA genes. The total AT content of the genome, as is typical for decapods, is 72.9%. We identified a non-coding control region of 327 bp according to its location and AT-richness. This is the smallest control region discovered in crustaceans so far. A mechanism of cytoplasmic tRNA import was addressed to compensate for the four missing tRNAs. The *S. crosnieri *mitogenome exhibits a novel arrangement of mitochondrial genes. We investigated the mitochondrial gene orders and found that at least six rearrangements from the ancestral pancrustacean (crustacean + hexapod) pattern have happened successively. The codon usage, nucleotide composition and bias show no substantial difference with other decapods. Phylogenetic analyses using the concatenated nucleotide and amino acid sequences of the 13 protein-coding genes prove consistent with the previous classification based upon their morphology.

**Conclusion:**

The present study will supply considerable data of use for both genomic and evolutionary research on hydrothermal vent ecosystems. The mitochondrial genetic characteristics of decapods are sustained in this case of *S. crosnieri *despite the absence of several tRNAs and a number of dramatic rearrangements. Our results may provide evidence for the immigrating hypothesis about how vent species originate.

## Background

Since intraorganellar DNA characteristics were found in chick embryo mitochondria [1,2], determination of mitochondrial genomes (mitogenomes) has become an important part of genome research. In 1981, Anderson *et al *reported the complete sequence of the human mitogenome [3]. This was the first identified organellar genome. Until recently, 1,212 metazoan mitogenomes have now been determined (NCBI Organelle Genome Resource [4]). With a few exceptions [5-9], animal mitogenomes always consist of the same 37 genes which incorporate 13 subunits of proteins involved in the respiratory chain and oxidative phosphorylation, two ribosomal RNAs (rRNAs), and 22 transfer RNAs (tRNAs) (reviewed in [10]). These sequences can provide large datasets for phylogenetic analyses at different levels also serving as ideal models of gene rearrangement and genome evolution.

152 arthropod mitogenomes have been determined since Clary and Wolstenholme sequenced that of *Drosophila yakuba *in 1985 [11]. Within the subphylum Crustacea, only 36 mitogenomes have been determined: one for each of the classes Cephalocarida, Ostracoda, Pentastomida and Remipedia, four for the Branchiopoda, eight for the Maxillopoda and 20 for the Malacostraca (including 14 decapods, see Table 1). Within the order Decapoda, the sampling is imbalanced: four for the suborder Dendrobranchiata and ten for the Pleocyemata (five for the infraorder Brachyura, two for the Caridea, and one for each of the Anomura, Astacidea and Palinura). Most decapod mitogenomes share the ancestral pancrustacean (crustacean + hexapod) gene order that shows only a *trnL-UUR *translocation from the ancestral arthropod arrangement depicted by the horseshoe crab *Limulus polyphemus *[12], or present only minor tRNA translocations. So far the anomuran mitogenome of the hermit crab *Pagurus longicarpus *has been determined to show dramatic gene rearrangements, including that of several large fragments [13].

**Table 1:** All decapod mitogenomes sequenced to date and their nucleotide compositions.

**Species**	**Suborder/Infraorder**	**Accession number**	**Length (bp)**	**Entire genome**			**Protein-coding gene**			***rrnL***		***rrnS***		**tRNAs**		**Control region**		**Reference**
										
				**AT%**	**GC-skew**	**AT-skew**	**Length (aa)**	**AT% (all)**	**AT% (3rd)**	**Length (bp)**	**AT%**	**Length (bp)**	**AT%**	**Length (bp)**	**AT%**	**Length (bp)**	**AT%**	
*Callinectes sapidus*	Brachyura	NC_006281	16263	69.1	-0.279	-0.011	3712	67.0	76.5	1323	71.8	785	70.3	1463	71.6	1435	78.2	[68]
*Portunus trituberculatus*	Brachyura	NC_005037	16026	70.2	-0.241	-0.051	3715	68.9	81.4	1332	73.8	840	70.1	1468	72.0	1104	76.4	[69]
*Pseudocarcinus gigas*	Brachyura	NC_006891	15515	70.5	-0.268	-0.006	3734	68.8	79.6	1324	74.9	821	73.8	1460	73.2	593	80.3	[70]
*Eriocheir sinensis*	Brachyura	NC_006992	16354	71.7	-0.248	-0.015	3718	68.9	79.5	1311	77.4	899	76.6	1473	72.4	896	83.1	[43]
*Geothelphusa dehaani*	Brachyura	NC_007379	18197	74.9	-0.341	-0.014	3711	71.5	83.4	1315	77.1	821	76.4	1519	75.8	514	87.2	[27]
*Pagurus longicarpus*^a^	Anomura	NC_003058	/	/	-0.213	+0.029	3698	69.6	84.0	1303	77.1	789	77.2	1458	74.1	/	/	[13]
***Shinkaia crosnieri*^b^**	**Anomura**	**EU420129**	**15182**	**72.9**	**-0.313**	**-0.014**	**3698**	**71.0**	**82.3**	**1331**	**77.8**	**811**	**78.1**	**1194**	**73.7**	**327**	**83.5**	**this study**
*Cherax destructor*	Astacidea	NC_011243	15895	62.4	-0.280	+0.029	3705	60.0	59.6	1302	67.9	917	68.3	1436	70.7	977	65.8	[28]
*Panulirus japonicus*	Palinura	NC_004251	15717	64.5	-0.182	-0.010	3715	62.6	66.8	1355	69.2	855	67.1	1484	68.9	786	70.6	[71]
*Halocaridina rubra*	Caridea	NC_008413	16065	63.2	-0.303	+0.055	3701	60.2	62.8	1351	68.2	872	68.8	1471	67.8	1020	78.4	[72]
*Macrobrachium rosenbergii*	Caridea	NC_006880	15772	62.3	-0.291	+0.151	3708	60.1	63.9	1305	66.0	852	66.0	1449	64.7	931	75.7	[70]
*Penaeus monodon*	Dendrobranchiata	NC_002184	15984	70.6	-0.136	-0.001	3716	69.3	83.7	1365	74.9	852	71.6	1494	68.0	991	81.5	[73]
*Marsupenaeus japonicus*	Dendrobranchiata	NC_007010	15968	66.5	-0.215	-0.018	3712	64.7	73.9	1367	70.5	853	67.9	1483	64.0	992	82.5	[74]
*Fenneropenaeus chinensis*	Dendrobranchiata	NC_009679	16004	68.9	-0.203	-0.011	3710	67.5	80.7	1367	72.7	852	69.9	1501	65.9	997	82.3	[47]
*Litopenaeus vannamei*	Dendrobranchiata	NC_009626	15990	67.8	-0.192	-0.026	3710	66.1	77.0	1371	71.8	853	69.6	1493	65.3	998	82.5	[47]

a Incomplete determination with ~300 bp of the control region unsequenced.

b Results of *Shinkaia crosnieri *in this study are given in bold

Hydrothermal vents were first discovered along the Galápagos Rift in 1977 [14]. Up to the present, their presence has been noted at mid-ocean spreading centers in the east Pacific, Atlantic, Arctic and Indian Oceans, and in the back-arc basins in the west Pacific (reviewed in [15]). These vent environments are considered as extreme given the high pressure, the high temperature (up to 390°C), the chemical toxicity of the fluids (H_2_S, CH_4 _and various heavy metals), and the total lack of photosynthetic production of animal nutrition. Nevertheless, with the exception of chemoautotrophic bacteria that oxidize hydrogen sulfide emitted from vents, surprisingly a number of specialized large faunas were also observed in these vents. Many invertebrates (e.g. vestimentiferan tube worms, vesicomyid and bathymodiolin bivalves, provannid gastropods, bythograeid and galatheid crabs, and bresiliid shrimps) often thrive with dramatically high densities, by utilizing (epi- or endo-)symbiotic chemoautotrophic bacteria or grazing on or filter feeding upon free-living chemoautotrophs [15]. There are two main hypotheses about how vent faunas originate. The relic hypothesis derives principally from morphological analyses of extant vent taxa [16,17], which considers the hydrothermal vent as a refugium for relic faunas during major historical extinction events. However, molecular studies of several vent-dominant taxa suggest that modern vent animals arose relatively recently [18]. An immigrating hypothesis seems more reasonable, which speculates that vent species may immigrate either from the non-vent environments or with close shallow-water relatives (reviewed in [15]).

Thanks to the effective Crustacea-specific versatile primers [19,20], mitochondrial DNA fragments can be used for studying the occurrence and dispersal of these vent species [21,22]. However, no entire mitogenome data has been available for any vent species to date. Baba and Williams first identified *Shinkaia crosnieri *at the Bismarck Archipelago and in the Okinawa Trough in the west Pacific Ocean [23]. The discoverers placed it into Decapoda: Anomura: Galatheidae according to its morphological features. Chan *et al *also mentioned this species as the first known hydrothermal crustacean in Taiwan [24]. Gathering near the top of the vents, *S. crosnieri *probably feed on polychaetes and "culture" filamentous bacteria on their abdominal surface [25]. No genetic data has been characterized for this species so far.

Here, we report the complete nucleotide sequence of the *S. crosnieri *mitogenome. It consists of the same 13 protein-coding genes, two rRNAs but only 18 tRNAs, with *trnS-UCN*, *trnW*, *trnC *and *trnY *missing from the usual structure. A mechanism of nuclear DNA-encoded tRNA import was addressed. Furthermore, the genes show surprising rearrangements from the ancestral pancrustacean order. Phylogenetic analyses indicate its close relationship with *P. longicapus*. No significant difference was found in the codon usage, nucleotide composition and bias. Our results may provide useful information on both genomics and the evolution of hydrothermal vent faunas.

## Results and Discussion

### Mitogenome organization

**Figure 1:**
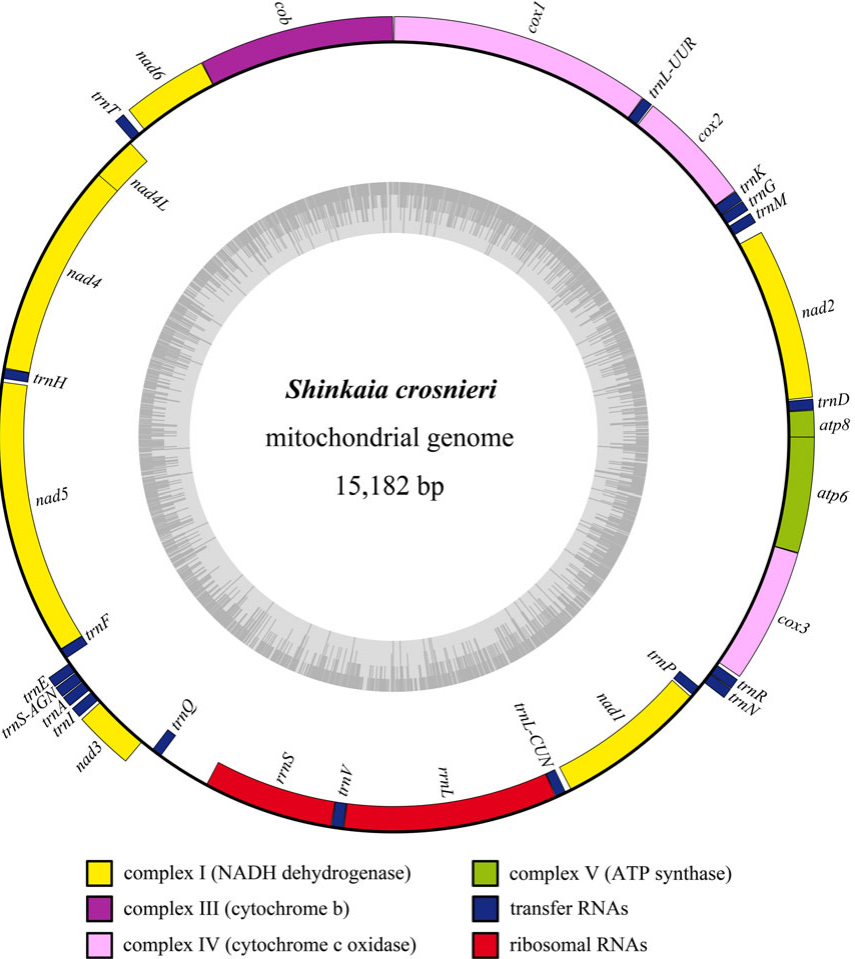
**The mitochondrial genome of *Shinkaia crosnieri***. Protein-coding genes, ribosomal and transfer RNA genes are presented as the Abbreviations section. The genes outside the circle are transcribed clockwise, while the genes inside are transcribed counterclockwise. Gene blocks are filled with different colors as the cutline shows. The inner ring indicates the GC content of the genome. The figure was initially generated with OGDRAW and modified manually.

The mitochondrial genome of the hydrothermal vent galatheid crab *Shinkaia crosnieri *is a 15,182-bp circular molecule (Figure 1). It is the smallest mitogenome found in the Malacostraca (15,289 [26] to 18,197 [27]) to date. The genome contains the same 13 protein-coding genes and two ribosomal RNAs as in most metazoans. However, it exhibits incomplete transfer RNA encoding (18 instead of the usual 22, see below). 22 mitochondrial genes are transcribed from one strand (the plus strand) and the remaining 11 from the other (the minus strand). Totally 740 non-coding nucleotides exist intergenically, with the largest continuous region (327 bp, AT% = 83.5) between *trnQ *and *rrnS*. Due to its location and AT-richness, we considered this part of the genome as a non-coding control region in the similar manner as in the case of the Australian freshwater crayfish *Cherax destructor *[28]. However, the control region is considerably smaller than those of any other decapods (Table 1) and shows no similarities with any previous decapod control region (data not shown). In the case of *Pagurus longicarpus *[13], approximately 300 bp of the control region remain unsequenced due to technical difficulties. Furthermore, as found in many mitogenomes, some genes overlap. *atp8*/*atp6 *and *nad4L*/*nad4 *each share seven nucleotides, although they are located on different reading frames. No further overlap of over two nucleotides was identified. Table 2 shows a summary of the *S. crosnieri *mitogenome organization.

**Table 2:** Gene contents of the mitochondrial genome of *Shinkaia crosnieri*.

**Gene**	**Strand^a^**	**Size (nt)**	**Start codon**	**Stop codon**	**Anticodon**	**Intergenic Nucleotides**
***cox1***	+	1531	GTT	T		0
***trnL-UUR***	+	65			TAA	5
***cox2***	+	688	ATG	T		0
***trnK***	+	67			TTT	1
***trnG***	+	64			TCC	18
***trnM***	+	65			CAT	61
***nad2***	+	1005	ATT	TAA		6
***trnD***	+	65			GTC	0
***atp8***	+	159	ATG	TAA		-7^b^
***atp6***	+	678	ATG	TAA		-1
***cox3***	+	792	ATG	TAA		17
***trnR***	+	67			TCG	1
***trnN***	+	65			GTT	39
***trnP***	+	66			TGG	12
***nad1***	-	939	ATG	TAA		32
***trnL-CUN***	-	67			TAG	0
***rrnL***	-	1331				0
***trnV***	-	76			TAC	0
***rrnS***	-	811				0
***control region***	+/-	327				0
***trnQ***	-	67			TTG	77
***nad3***	+	354	ATT	TAA		16
***trnI***	+	66			GAT	16
***trnA***	+	65			TGC	3
***trnS-AGN***	+	68			TCT	4
***trnE***	+	66			TTC	33
***trnF***	-	67			GAA	-2
***nad5***	-	1712	ATA	TA		18
***trnH***	-	64			GTG	0
***nad4***	-	1339	ATG	T		-7
**nad4L**	-	282	ATA	TAA		15
***trnT***	+	64			TGT	36
***nad6***	+	504	ATT	TAA		-1
***cob***	+	1141	ATG	T		3

a Plus strand (+)/minus strand (-)

b Negative values represent overlapping nucleotides.

### Protein-coding genes and ribosomal RNAs

For the protein-coding genes, nine (*atp8*, *atp6*, *cob*, *cox1*—*3*, *nad2*—*3 *and *nad6*) are encoded in the plus strand while the remaining four (*nad1*, *nad4–5 *and *nad4L*) in the minus strand (Figure 1; Table 2). This orientation is shared by all decapod mitogenomes sequenced to date. 12 out of the 13 protein-coding genes appear to start with the codon ATN (Table 2), typical for metazoan mitogenomes [29]. The *cox1 *initiates with GTT, in a case identical to *Neocalanus cristatus *[30]. Eight genes possess TAA as their termination codons (Table 2). Truncated termination codons (TA or T) are observed in *cox1*—*2*, *nad4*—*5 *and *cob*. Post-transcriptional polyadenylation can subsequently generate mature TAA codons [31].

The *rrnL *and *rrnS *genes of *S. crosnieri *are 1,331 (AT% = 77.8) and 811 bp (AT% = 78.1) in length, respectively. The lengths are among values typical for crustaceans whereas the AT contents are slightly higher than those of other decapod counterparts (Table 1).

### Transfer RNAs

**Figure 2:**
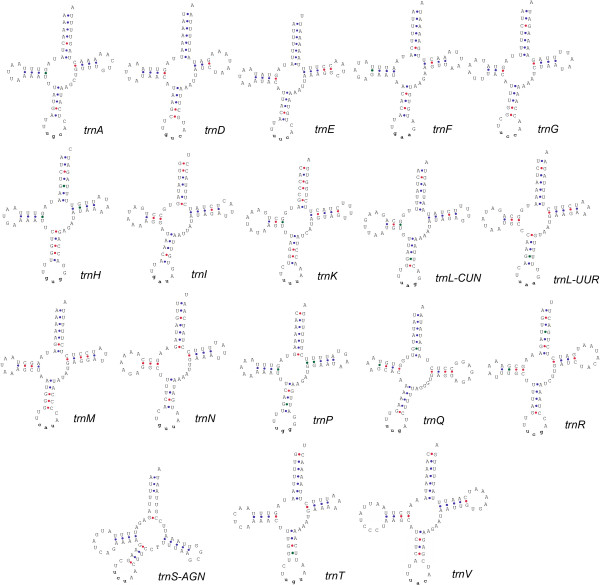
**Putative secondary structures for the 18 transfer RNAs of the *Shinkaia crosnieri *mitogenome**. 17 structures were generated by tRNAscan-SE and the rest *trnS-AGN *was folded manually. CG, AU and GU bonds are denoted by red, blue and green colors, respectively. The lowercase triplets indicate anticodons.

We analyzed the entire sequences of the genome and successfully identified 18 tRNA genes by their potential secondary structures (Figure 2). tRNAscan-SE determined 17 of them. The only manually folded *trnS-AGN *exhibits three mismatches on the acceptor stem while its DHU, TΨC and anticodon stems appear intact and well paired. The TΨC arm of *trnS-AGN *is often extremely short or completely missing in arthropods [32-35]. Besides *trnS-AGN*, each of *trnI*, *trnK *and *trnL-CUN *bears a mismatch on the acceptor stem. Usually tRNAs with a U in the wobble position (the first position) of the anticodons recognize either four-fold degenerate or NNR codons; those with a G in this position only recognize NNY codons. All tRNAs of *S. crosnieri *mitogenome obey this rule except for *trnM*, whose anticodon CAU recognizes both ATG and ATA. The C can be post-transcriptionally modified to 5-formylcytidine to pair with the ATA codon [36].

*trnS-UCN*, *trnW*, *trnC *and *trnY *are missing from the *S. crosnieri *mitogenome. We analyzed all fractions between protein-coding or ribosomal RNA genes [see Additional file 1], but no extra tRNAs or similar sequences were identified in the genome. This is not an exception in arthropods considering the absence of *trnQ *in the mitogenome of the whitefly *Aleurodicus dugesii*, *trnS-AGN *in the aphid *Schizaphis graminum *[37], and *trnD *in the scorpion *Centruroides limpidus *[7]. Deficiencies of tRNA genes were often observed in protozoans, fungi, algae, plants and low metazoans (reviewed in [38]). In these cases, the mechanism of nuclear DNA-encoded tRNA import proves responsible for mitochondrial tRNA compensation. Furthermore, marsupial mitochondrial tRNAs show interesting patterns. Janke and Paabo identified a pseudogene-like *trnD *in *Didelphis virginiana*, as its anticodon is GCC instead of the usual GUC [39]. They found that the cytosine is changed to uridine under a post-transcriptional RNA editing. Dorner *et al *discovered a *trnK *pseudogene in the same marsupial species by observing its non-functional secondary structure [40]. Cytoplasmic tRNA import rather than RNA editing solves this problem. In our study, there seems no candidate template for RNA editing. The mechanism of tRNA import is therefore a plausible explanation.

### Nucleotide composition and codon usage

The nucleotide composition of *S. crosnieri *mitogenome is as follows: A = 5,452 (35.9%), G = 1,415 (9.3%), T = 5,612 (37.0%) and C = 2,703 (17.8%). The genome has an overall AT content of 72.9%, which appears high for decapods (62.3–74.9%), but is a little lower than that of *Geothelphusa dehaani *[27]. The composition is strongly skewed away from G in favor of C (the GC-skew is -0.313) while almost balanced for A and T (the AT-skew is -0.014). This feature is well conserved within decapods (Table 1). In mammals, the duration of single-stranded state of the "heavy-stranded" genes during mitochondrial DNA replication can explain this asymmetry [41]. Whether or not the same explanation works on our results remains difficult to predict at this time due to the scarcity of information regarding DNA replication of invertebrate mitochondria.

The *S. crosnieri *mitogenome totally encodes 3,698 amino acids of protein-coding genes. Table 3 shows the codon usage. Although four tRNAs are absent, the corresponding codons 235 TCNs, 95 TGRs, 42 TGYs and 146 TAYs (for *trnS-UCN*, *trnW*, *trnC *and *trnY*, respectively) are used quite frequently (14% of total codons), evidently raising the need for tRNA compensation. We observed a strong AT-bias (AT% = 82.3) in the third codon positions, similar to those in the Anomura relative *P. longicarpus*, the Brachyura and the suborder Dendrobranchiata (73.9–84.0%). The values in the Caridea, Palinura and Astacidea are relatively lower (59.6–66.8%).

**Table 3:** Codon usage in protein-coding genes of *Shinkaia crosnieri*.

**Phe**	TTT	325	**Ser^a^**	TCT	125	**Tyr^a^**	TAT	119	**Cys^a^**	TGT	36
	TTC	36		TCC	32		TAC	27		TGC	6
**Leu**	TTA	341		TCA	73	**END^b^**	TAA	8	**Trp^a^**	TGA	78
	TTG	38		TCG	5		TAG	0		TGG	17
**Leu**	CTT	101	**Pro**	CCT	58	**His**	CAT	50	**Arg**	CGT	17
	CTC	42		CCC	45		CAC	30		CGC	6
	CTA	49		CCA	37	**Gln**	CAA	60		CGA	31
	CTG	5		CCG	3		CAG	11		CGG	4
**Ile**	ATT	328	**Thr**	ACT	66	**Asn**	AAT	117	**Ser**	AGT	35
	ATC	46		ACC	19		AAC	30		AGC	5
**Met**	ATA	193		ACA	60	**Lys**	AAA	81		AGA	80
	ATG	40		ACG	5		AAG	16		AGG	25
**Val**	GTT	88	**Ala**	GCT	81	**Asp**	GAT	54	**Gly**	GGT	65
	GTC	9		GCC	46		GAC	9		GGC	14
	GTA	80		GCA	42	**Glu**	GAA	62		GGA	112
	GTG	18		GCG	4		GAG	19		GGG	42

a The corresponding tRNAs are missing in the mitogenome.

b Termination codons. The incomplete TA/T are not included.

### Gene rearrangements

**Figure 3:**
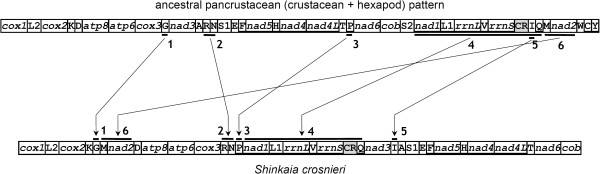
**Gene rearrangements of the *Shinkaia crosnieri *mitogenome**. At least six rearrangements happen between mitogenomes of *S. crosnieri *and the ancestral pancrustacean pattern: (1) *trnG*; (2) *trnR-trnN*; (3) *trnP*; (4) *nad1-trnL-CUN-rrnL-trnV-rrnS*-CR-*trnQ*; (5) *trnI*; and (6) *trnM-nad2*. All tRNAs are designated by single letters (except L1, L2, S1 and S2 for *trnL-CUN*, *trnL-UUR*, *trnS-AGN *and *trnS-UCN*, respectively). Genes encoded on the minus strand are underlined. The putative control regions (CRs) are shaded.

The *S. crosnieri *mitogenome shows a novel arrangement within arthropods. The gene order diverges in many positions from that of the ancestral pancrustacean pattern shared by lots of crustaceans and hexapods [42]. Totally, we identified at least six rearrangements in this species (Figure 3). Two rearrangements involve protein-coding genes while the remainders are tRNA translocations. A major fragment containing *nad1*, *trnL-CUN*, *rrnL*, *trnV*, *rrnS*, control region and *trnQ *moves to upstream of *nad3 *from its ancestral position; *trnI *may translocate before or after this event. The fraction *trnM*-*nad2 *moves to upstream of *trnD*. This event, together with the *trnG *translocations, indicates potential synapomorphic characters for the Anomura. The location of *trnR*-*trnN *changes to downstream of *cox3*. The *trnP *moves to downstream of *nad1*. *P. longicarpus *[13] and *Eriocheir sinesis *[43] share this rearrangement. Finally, the *trnI *moves to the middle of *nad3 *and *trnA*. This translocation is novel within crustaceans. Rearrangements of *trnI *were observed in *Speleonectes tulumensis *[44] and *Tigriopus japonicus *[45], where *trnI *is translocated to different positions in both species. Morrison *et al *inferred parsimonious rearrangements of the crab-like form [46]. They pointed out that the Galatheoidea diverged after the translocations of *trnQ *and *trnI-trnM-nad2*. Our results support their hypothesis.

**Figure 4:**
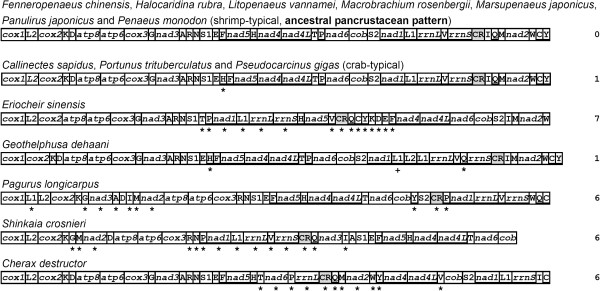
**Gene rearrangements of decapod mitogenomes**. The ancestral pancrustacean pattern is depicted by four dendrobranchiatans, two carideans and one palinuran. All tRNAs are designated by single letters (except L1, L2, S1 and S2 for *trnL-CUN*, *trnL-UUR*, *trnS-AGN *and *trnS-UCN*, respectively). Asterisks indicate rearrangement from the ancestral order. A cross shows the insertion of a *trnL-CUN *in *Geothelphusa dehaani*. Putative control regions (CRs) are shaded. Genes encoded on the minus strand are underlined. Numbers on the right of each genome specify block interchanges from the ancestral pattern. Especially, in the mitogenome calculations on *G. dehaani *and *Shinkaia crosnieri*, the additional or absent genes were excluded respectively.

**Figure 5:**
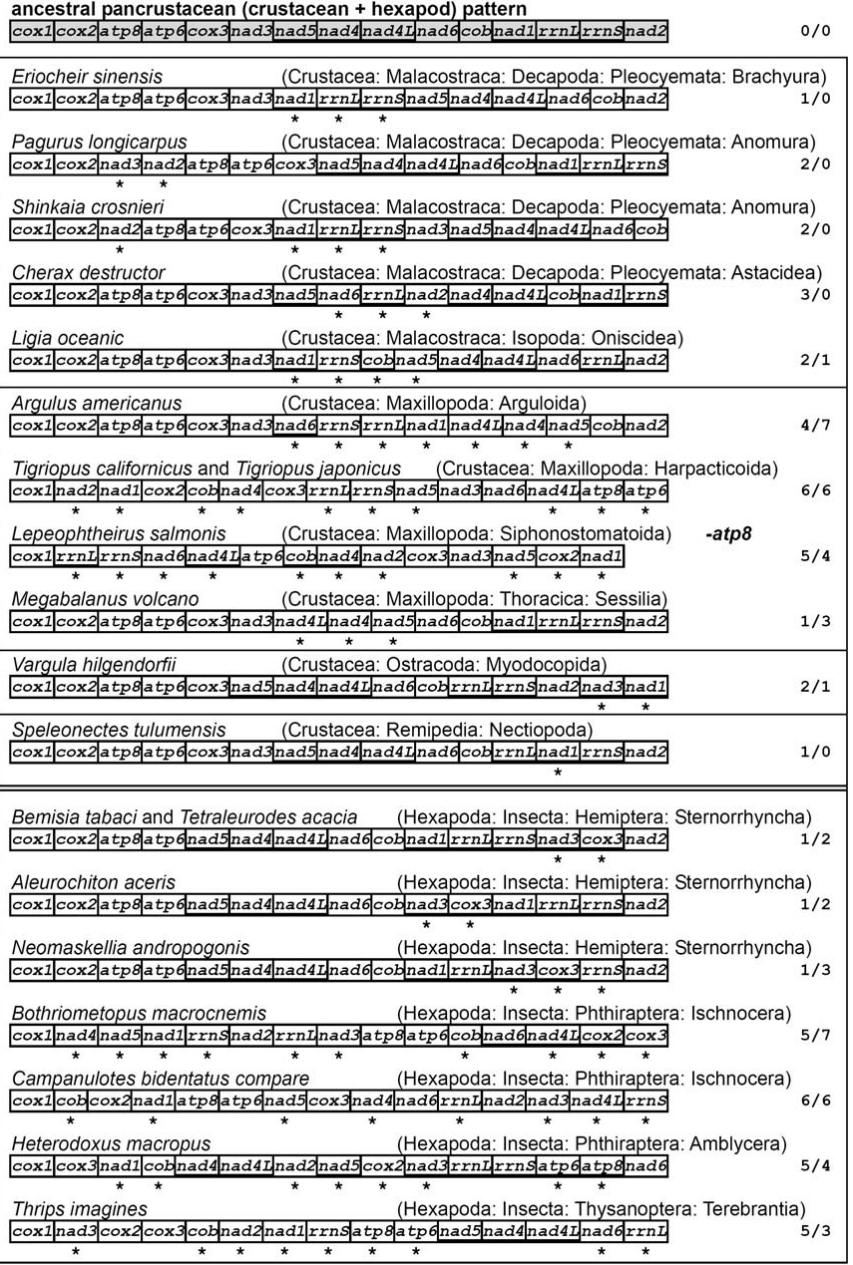
**Large block rearrangements of pancrustacean mitogenomes**. All tRNAs and control region (CR) are removed from each mitogenome. The ancestral pancrustacean pattern is shaded. Asterisks indicate the different positions or orientations from the ancestral genome. Genes encoded on the minus strand are underlined. Numbers on the right of each genome specify block interchanges/inversions. The taxonomy is showed as subphylum: class: order (: suborder: infraorder) in brackets. The Crustacea and Hexapoda are separated with a double line while classes are divided by single lines. The gene *atp8 *is absent in the *Lepeophtheirus salmonis *mitogenome and is therefore excluded from the calculation.

We also analyzed data gathered thus far on rearrangements of both decapod (Figure 4) and all pancrustacean mitogenomes (Figure 5). For simplicity of analysis, all tRNAs and control regions (CRs) were excluded in the case of the pancrustaceans. The *E. sinensis *mitogenome shows the maximum 7 block interchanges within the Decapoda, and *Shinkaia crosnieri *exhibits 6 interchanges from the ancestral pancrustacean arrangement on its mitogenome (Figure 4). The ancestral pancrustacean pattern depicted by four dendrobranchiatans, two carideans and one palinuran, has only a *trnL-UUR *translocation from the ancestral arthropod order of *Limulus polyphemus *[12]. In the analyses of large block (protein-coding genes plus rRNAs) rearrangements of all pancrustaceans, the class Maxillopoda shows maximum inversions (3–7) within crustaceans. In the case of the Hexapoda (81 mitogenomes determined to date), all large block rearrangements have happened within the class Insecta (Figure 5), where more rearrangements (both translocations and inversions) were observed within the orders Phthiraptera and Thysanoptera.

### Phylogenetic analysis

**Figure 6:**
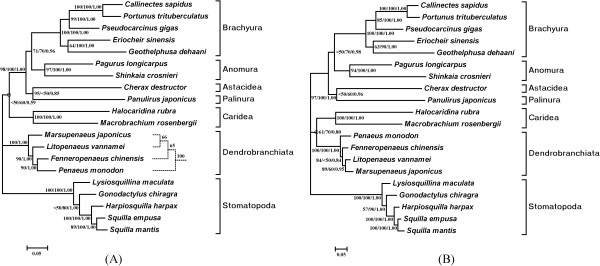
**Phylogenetic trees of decapod relationships from the nucleotide (A) and amino acid datasets (B)**. Five stomatopods served as outgroups. Branch lengths and topologies came from the Bayesian analyses. Numbers besides the nodes specify bootstrap percentages from maximum parsimony (MP) and maximum likelihood (ML) plus Bayesian posterior probabilities (BPP). Especially, nodes inside the Dendrobranchiata clade in (A) are noted as ML/BPP, with a different dashed subtree from MP on the right. The nodes with open circles receive modest statistical support (MP < 75, ML < 75 and BPP < 0.95).

We used both nucleotide and amino acid sequences of protein-coding genes for the intraorder phylogenetic analyses. For each dataset, the maximum likelihood (ML) and Bayesian analysis gave the same tree topology (Figure 6) while the maximum parsimony (MP) analysis of the nucleotides showed a minor difference (Figure 6A). The nucleotide and amino acid trees are nearly the same except for the position of the Caridea clade (*Halocaridina rubra *+ *Macrobrachium rosenbergii*) and the inner structure of the Dendrobranchiata clade. In the case of the nucleotides, the clade is placed together with other pleocyematans (Figure 6A), but not well supported (MP/ML/BPP = <50/60/0.59). However, in the amino acid tree, the Caridea shows a sister position of the Dendrobranchiata (Figure 6B), which also remains modestly statistically supported (MP/ML/BPP = 61/70/0.80). Therefore, according to our results, whether the Pleocyemata is monophyletic or paraphyletic appears ambiguous. The Dendrobranchiata clade was well reconstructed in the ML and Bayesian analyses of the nucleotide dataset (Figure 6A) but unresolved in the case of the MP analysis of the nucleotides and in all three analyses of the amino acids (Figure 6). The relationship (*Marsupenaeus *+ (*Litopenaeus *+ (*Fenneropenaeus *+ *Penaeus*))) is more believable, as discussed in [47]. In both trees, the two anomurans (*S. crosnieri *and *P. longicarpus*) form a separate clade, according with the relationship derived from the morphology [23]. Whether or not the Anomura is monophyletic needs intensive sampling. In fact, phylogenetic relationships within this infraorder remain largely unsettled (reviewed in [48]). Furthermore, no unexpectedly long or short branch length of *S. crosnieri *was observed (Figure 6), indicating that the evolutionary acceleration/retardation is probably not evident for this vent species. Accurate calculation on evolutionary rates needs fossil records that remain absent for these vent taxa [49].

## Conclusion

This is the first determination of the mitochondrial genome of a hydrothermal vent organism. It not only enlarges sampling with the Crustcean, but more importantly, provides useful information for understanding the evolutionary history of vent species.

The mitogenome of *Shinkaia crosnieri *exhibits almost the same characteristics with those of other metazoans with the exception of two differences. Firstly, four transfer RNAs are missing from the mitogenome. The codons recognized by these four tRNAs are frequently used (14%) for protein encoding. We proposed a mechanism of nuclear DNA-encoded tRNA import for this compensation. Secondly, the arrangement of the gene contents is significantly different from those of other pancrustacen mitogenomes. The changes are complicated and at least six rearrangements were observed. Some of them are shared by other crustaceans. However a novel *trnI *translocation was identified.

The codon usage, nucleotide composition and bias of *S. crosnieri *mitogenome are similar with other decapods. Phylogenetic analysis shows a close relationship with another anomuran *Pagurus longicarpus*, supporting for the taxonomic classification by the morphology [23]. No trend of evolutionary acceleration or retardation was observed. An exterior origin may explain these conserved features found in *S. crosnieri*. In fact, one galatheid, *Munidopsis subsquamosa*, seems to be a non-vent species which can invade vent habitats for at least short periods of time [50]. Our results may provide some evidence for the immigrating hypothesis about how hydrothermal vent species originate.

## Methods

### Samples and DNA preparation

One specimen of *Shinkaia crosnieri *was a generous gift from the Japan Agency for Marine-Earth Science and Technology (JAMSTEC). Sampling was done using manipulators and a slurp gun with single or six canisters set on the ROV Hype-dophin of JAMSTEC on Apr 24, 2005. The hydrothermal vent galatheid crab was collected at the Jade Site of the Izena Hole (27°16.307' N, 127°4.884' E, 1,309 m) in the Okinawa Trough.

150 mg of muscle was dissected in 1.2 ml of extraction buffer (60 mM Tris-HCl [pH 8.0], 100 mM EDTA and 0.5% SDS). Proteinase K was added to a final concentration of 500 μg/ml. Samples were digested thoroughly at 55°C. DNA was precipitated from the supernatant by the phenol/chloroform method [51] and dissolved in TE buffer (10 mM Tris-HCl [pH 7.4] and 1 mM EDTA).

### Genome determination

Partial genic fragments of the genes cytochrome oxidase subunit 1 (*cox1*), large ribosomal RNA (*rrnL*) and NADH dehydrogenase subunit 5 (*nad5*) were amplified using the primers LCO1490/HCO2198 [19], 16Sar/16Sbr [20], and DEnad5F/DEnad5R, respectively [see Additional file 2]. The reactions were carried out with 1.25 units of *Taq *DNA polymerase (Promega, Madison, USA) and 125 ng of genomic DNA as templates, according to the manufacturer's instructions. The amplified fragments were cloned into pUCm-T vectors (Sangon, Shanghai, China) that were sequenced using the versatile primers M13F/R. After determination, the partial sequences were used for designing gene-specific primers. To facilitate the subsequent long PCR [52], we chose candidates with high melting temperatures. Long PCRs were performed with 1.25 units of LA *Taq *DNA polymerase (TaKaRa, Shiga, Japan) following the manufacturer's commendations. For each long fragment, we used nested primer pairs [see Additional file 2] and a two-step cycle (denaturation and annealing/extension). Considering the lack of available genome arrangement data for this species, all primer combinations were attempted. The long fragments were bidirectionally sequenced using a primer-walking strategy. All amplifications were done on a TGradient thermocycler (Whatman-Biometra, Goettingen, Germany). All sequencing was performed with ABI PRISM Big Dye terminator chemistry and analyzed on ABI 3730 automated sequencers (Applied Biosystems, Foster City, USA).

### Gene annotation and sequence analysis

All sequences were edited using EditSeq version 5.00 (DNAStar, Madison, USA) and Vector NTI version 9.0.0 (InforMax, Frederick, USA). Protein-coding and ribosomal RNA genes were identified by aligning with other decapod mitochondrial genomes (Table 1). Borders of rRNA genes were determined by adjacent genes. Transfer RNAs were initially detected by tRNAscan-SE version 1.21 [53] while the rest were identified by their potential secondary structures and anticodons. A circular display of *S. crosnieri *mitochondrial genome was depicted by OGDRAW version 1.1 [54] and modified manually. All annotation information was imported to Sequin version 7.90 (NCBI) in which generated a standard output of submitted data [see Additional file 3]. The nucleotide sequences are deposited in GenBank/EMBL/DDBJ under the accession number EU420129. Codon usage and nucleotide composition were analyzed with MEGA 4 [55]. Strand skew values were calculated according to the formulae by Perna and Kocher [56].

### Rearrangement analysis

Block interchanges are defined as the minimum rearrangements from one genome to another. We used the ROBIN website [57] for calculating block interchanges of each mitogenome deviated from the ancestral pancrustacean (crustacean + decapod) arrangement. For decapod mitogenomes, we used all 37 genes plus the control region (CR). Considering the complication and inconstancy of their translocations, tRNAs and CR were omitted from the calculation on all pancrustacean rearrangements. Inversions were directly identified by gene orientations.

### Phylogenetic analysis

For phylogenetic analysis, we chose the 14 other decapod mitochondrial genomes determined to date (Table 1). The five stomatopods *Gonodactylus chiragra *[GenBank:NC_007442], *Harpiosquilla harpax *[NC_006916], *Lysiosquilla harpax *[NC_007443], *Squilla empusa *[NC_007444] and *Squilla mantis *[NC_006081] served as outgroups. Both nucleotides and amino acids of 13 protein-coding genes were subjected to concatenated alignments using ClustalX version 1.81 [58], with the opening/extension gap penalties 15/6.66 and 10/0.2, respectively. For the nucleotides, we omitted the third position of the codons before the alignment, according to the result of a saturation analysis [59] by DAMBE version 4.5.57 [60]. Ambiguously aligned proportions of both alignments were excluded using Gblocks version 0.91b [61] with default block parameters. The final nucleotide and amino acid datasets consisted of 7,027 nt and 3,340 aa, respectively.

For the nucleotide dataset MODELTEST version 3.7 [62] selected the model GTR+G+I for both the likelihood and Bayesian analyses. ProtTest version 1.4 [63] chose the model MtArt+G+I for the amino acid dataset. Both selections were done following the Akaike information criterion (AIC) [64]. As MtArt is so recent a model for the arthropod evolution [65], we could not implement it in the Bayesian analysis for the amino acids, where we used the best scoring alternative MtRev+G+I.

Maximum parsimony (MP) analyses with both datasets were done using a close-neighbor-interchange (CNI) with search level 1 method implemented in MEGA 4 [55], with 1,000 bootstrap replicates.

We performed maximum likelihood (ML) analyses of both nucleotide and amino acid alignments using TREEFINDER version of Mar, 2008 [66] under the models GTR+G+I and MtArt+G+I, respectively. The alpha shape parameters were estimated from the datasets. The analyses started from BioNJ trees. Branch statistical supports were obtained after 100 bootstrap replicates each.

Bayesian analyses of both nucleotide and amino acid alignments were carried out using MRBAYES version 3.1.2 [67], under the models GTR+G+I and MtRev+G+I (see above), respectively. The Markov Chain Monte Carlo analyses were performed for 1,000,000 generations in two runs of eight chains each. The Bayesian posterior probability (BPP) of each tree partition was estimated by sampling the trees every 1,000 generations after discarding the first 10%.

## Abbreviations

Mitochondrial genes: *atp6 and atp8*: ATP synthase subunits 6 and 8; *cob*: cytochrome b; *cox1–3*: cytochrome c oxidase subunits 1–3; *nad1–6 *and *nad4L*: NADH dehydrogenase subunits 1–6 and 4L; *rrnS *and *rrnL*: small and large subunit ribosomal RNA (rRNA) genes; *trnX*: transfer RNA (tRNA) genes, where *X *is the one-letter abbreviation of the corresponding amino acid; CR: putative control region; MP: maximum parsimony; ML: maximum likelihood; BPP: Bayesian posterior probability; aa: amino acids; nt: nucleotides; bp: base pairs.

## Authors' contributions

J–SY designed and performed the experiments, analyzed the data, and wrote the paper. W–JY directed the research and in collaboration with J–SY performed the data analysis and paper writing.

## Supplementary Material

Additional file 1 Supplementary Table 1 – *Analyses of intergenic fractions of the *Shinkaia crosnieri *mitochondrial genome*. Click here for file

Additional file 2 Supplementary Table 2 – *Primers for determination of the *Shinkaia crosnieri *mitochondrial genome*. Click here for file

Additional file 3 *The entire *Shinkaia crosnieri *mitochondrial genome (GenBank format)*. Click here for file
