# The complete mitochondrial genome sequence of the hydrothermal vent galatheid crab *Shinkaia crosnieri *(Crustacea: Decapoda: Anomura): A novel arrangement and incomplete tRNA suite

**DOI:** 10.1186/1471-2164-9-504

**Published:** 2008-10-27

**Authors:** Jin-Shu Yang, Hiromichi Nagasawa, Yoshihiro Fujiwara, Shinji Tsuchida, Wei-Jun Yang

**Affiliations:** 1 Institute of Cell Biology and Genetics, College of Life Sciences, Zijingang Campus, Zhejiang University, Hangzhou, Zhejiang 310058, PR China; 2 State Conservation Center for Gene Resources of Wildlife and the Key Laboratory of Conservation Genetics and Reproductive Biology for Wild Animals of the Ministry of Education, Hangzhou, Zhejiang 310058, PR China; 3 Department of Applied Biological Chemistry, Graduate School of Agricultural and Life Sciences, The University of Tokyo, Tokyo 113-8657, Japan; 4 Extremobiosphere Research Center, Japan Agency for Marine-Earth Science and Technology (JAMSTEC), 2-15 Natsushima-cho, Yokosuka, Kanagawa 237-0061, Japan

## Correction

After publication of this work [1], we found that we inadvertently included an incomplete list of all co-authors. A full list of authors and affiliations including Hiromichi Nagasawa, Yoshihiro Fujiwara and Shinji Tsuchida has been added.

Accordingly, some parts in "Samples and DNA preparation" of Methods, Authors' contributions and Acknowledgements have been revised as follows:

## Samples and DNA preparation

One specimen of *Shinkaia crosnieri *was used for the experiments. Sampling was conducted using a suction sampler with multiple canisters installed on the ROV Hyper-Dophin belonging to the Japan Agency for Marine-Earth Science and Technology (JAMSTEC) on Apr 24, 2005. *[The followings are the same.]*

## Authors' contributions

J-SY designed and performed the experiments, analyzed the data, and wrote the paper. HN participated in DNA preparation. YF and ST collected the materials. W-JY directed the research and paper writing. All authors contributed to the manuscript and then read and approved the final version.
