# Supplementary material for: The complete mitochondrial genome sequence of the hydrothermal vent galatheid crab Shinkaia crosnieri (Crustacea: Decapoda: Anomura): A novel arrangement and incomplete tRNA suite
Source: BMC Genomics. 2008 May 30;9:257. doi: 10.1186/1471-2164-9-257 (PMC2442616; doi:10.1186/1471-2164-9-257)
Supplement: Additional file 1 — Supplementary Table 1 – Analyses of intergenic fractions of the Shinkaia crosnieri mitochondrial genome. [file 1471-2164-9-257-S1.doc]

**Supplementary Table 1 – Analyses of intergenic fractions of *Shinkaia crosnieri* mitochondrial genome^a^**

| ***cox1*** |  | ***cox2*** |  |  |  | ***nad2*** |  | ***atp8*** | ***atp6*** | ***cox3*** |  |  |  | ***nad1*** |  | ***rrnL*** |  | ***rrnS*** |  |  | ***nad3*** |  |  |  |  |  | ***nad5*** |  | ***nad4*** | ***nad4L*** |  | ***nad6*** | ***cob*** |
| --- | --- | --- | --- | --- | --- | --- | --- | --- | --- | --- | --- | --- | --- | --- | --- | --- | --- | --- | --- | --- | --- | --- | --- | --- | --- | --- | --- | --- | --- | --- | --- | --- | --- |

| **Intergene**  **(length)** | **Primer** | **Location^b^** | **Sequences (5’->3’)** |
| --- | --- | --- | --- |
| *cox1-cox2* | SCcox1F | 1309-1334 | TACTCAGATTATCCAGATGCCTACAC |
| (326 bp) | SCcox2R | 1609-1634 | ATCTTGAAAATTTAAGTAACCTCATG |
| *cox2-nad2* | SCcox2F | 2107-2130 | GTATTAAAGCAGATGCTATCCCCG |
| (564 bp) | SCnad2R | 2647-2670 | TTCAAGGCCAGTTCATACACTAAA |
| *nad2-atp8* | SCnad2F | 3289-3308 | TCTTTAGGAGGATTCCCCCC |
| (471 bp) | SCatp8R | 3735-3759 | AGGTTGAAATTATTTTTTTTGGTTG |
| *atp8-atp6* | SCatp8F | 3654-3675 | GGCCCCATTTTATGACTAAACC |
| (273 bp) | SCatp6R | 3905-3926 | AAGATCAACGTGAGGGAGAAGC |
| *atp6-cox3* | SCatp6F | 4422-4441 | CCTATGTATTTGCTGTTCTA |
| (255 bp) | SCcox3R | 4656-4676 | TAGGTTCCTTCTCGTGTAGTA |
| *cox3-nad1*^c^ | SCcox3F | 5127-5146 | ATGTCTTTACCGTCTTTACT |
| (547 bp) | SCnad1F | 5654-5673 | TTTATTTGAGTGCGAGGAAC |
| *nad1-rrnL* | SCnad1R | 6404-6429 | AAAGCCACTCCAACTAATACACATAA |
| (263 bp) | SCrrnLF | 6643-6666 | CGTAAGCCAGGTTGGTTTCTATCT |
| *rrnL-rrnS* | SCrrnLR | 7789-7813 | TTTACTCTAACCTCTTATTCTATTT |
| (215 bp) | SCrrnSF | 7982-8003 | TAGGTGTACTGGAAAGTTTACC |
| *rrnS-nad3* | SCrrnSR | 8565-8589 | CTAGCTATTTATGTATAACCGCAGC |
| (800 bp) | SCnad3R | 9340-9364 | ATTTTTTTCTCGATCTTTGTTTGTT |
| *nad3-nad5* | SCnad3F | 9573-9593 | ATGAATGAAATCAAGGAGCCC |
| (704 bp) | SCnad5F | 10252-10276 | ATATGAATCTAGTTTTTTTGGTGGA |
| *nad5-nad4* | SCnad5R | 11428-11449 | AAAGATCCCCATAGATCCTACG |
| (479 bp) | SCnad4F | 11885-11906 | TGTTGTTCTGGAAAAGTCCGAG |
| *nad4-nad4L* | SCnad4R | 12799-12821 | CGGCCCTCAAAATTAATATAAAA |
| (591 bp) | SCnad4LF | 13366-13389 | GAGGTTACAGGGGATTAGTAGCAT |
| *nad4L-nad6* | SCnad4LR | 13199-13222 | TACCCAAAATGATAAACCTAGGGC |
| (406 bp) | SCnad6R | 13573-13596 | CGGATAGAGGGTGGATTATACTTA |
| *nad6-cob* | SCnad6F | 14002-14025 | TAAACACTTCTTCTAGTCCCCTCC |
| (358 bp) | SCcobR | 14336-14359 | TGAACCATAGTATACTCCTCGTCC |
| *cob-cox1* | SCcobF | 14814-14841 | CTGCTAATCCTTTAGTAACACCTACCCA |
| (727 bp) | SCcox1Ri | 333-358 | CAGCACCATTTTCCACTATTCCTCTT |

a The genome is linearized with blanks as tRNAs. The control region is shaded.

b Nucleotides numbering from *cox1*.

c Genes coding from the minus strand are underlined.
