# Supplementary material for: The complete mitochondrial genome sequence of the hydrothermal vent galatheid crab Shinkaia crosnieri (Crustacea: Decapoda: Anomura): A novel arrangement and incomplete tRNA suite
Source: BMC Genomics. 2008 May 30;9:257. doi: 10.1186/1471-2164-9-257 (PMC2442616; doi:10.1186/1471-2164-9-257)
Supplement: Additional file 2 — Supplementary Table 2 – Primers for determination of the Shinkaia crosnieri mitochondrial genome. [file 1471-2164-9-257-S2.doc]

**Supplementary Table 2 - Primers for determination of *Shinkaia crosnieri* mitochondrial genome**

| **PCR**  **amplifications** | **Primer name** | **Primer sequences (5’->3’)** | **Location^a^** | **Product^b^** | **Annealing temperature** |
| --- | --- | --- | --- | --- | --- |
| **partial PCRs** | **LCO1490** | GGTCAACAAATCATAAAGATATTGG | 14-38 | 709 bp | 45 ^o^C |
|  | **HCO2198** | TAAACTTCAGGGTGACCAAAAAATCA | 697-722 |  |  |
|  | **16Sar** | CGCCTGTTTATCAAAAACAT | 7200-7219 | 553 bp | 45 ^o^C |
|  | **16Sbr** | CCGGTCTGAACTCAGATCACGT | 6667-6688 |  |  |
|  | **DEnad5F** | TATGTGGDWTWCCTTTTWTAGCDGG | 10664-10688 | 275 bp | 52 ^o^C |
|  | **DEnad5R** | ATHTCAAGMTAARCHAGCHCCHCC | 10414-10437 |  |  |
| **long PCRs,**  **1st round** | **SCcox1Fo** | ATAAGAGGAATAGTGGAAAATGGTGCTG | 331-358 | / | 66 ^o^C^b^ |
|  | **SCrrnLFo** | TAAATAGAGTCTAACCTGCCCACTGAAT | 7159-7186 |  |  |
|  | **SCrrnLRo** | CCTCTGTTTTCATTTTAAGACAGTTTCC | 6867-6894 |  |  |
|  | **SCnad5Fo** | AACGATGAGAGGCGAATAATAACT | 10474-10497 |  |  |
|  | **SCnad5Ro** | AGTTATTATTCGCCTCTCATCGTT | 10474-10497 |  |  |
|  | **SCcox1Ro** | GGGAGAGATAAAAGTAAAAGGATGGC | 565-590 |  |  |
| **long PCRs,**  **2nd round** | **SCcox1Fi** | CAGCCATCCTTTTACTTTTATCTCTCCC | 563-590 | 6425 bp | 66 ^o^C^b^ |
|  | **SCrrnLFi** | TTTGGAAACTGTCTTAAAATGAAAACA | 6871-6987 |  |  |
|  | **SCrrnLRi** | TTTCTTTGTTCAACCATTCATTCAAG | 7067-7092 | 3381 bp |  |
|  | **SCnad5Fi** | TTCTGTGTTTGGAGGTGCTTTAT | 10425-10447 |  |  |
|  | **SCnad5Ri** | CGCCTCTCATCGTTGATATTTCTT | 10484-10507 | 5057 bp |  |
|  | **SCcox1Ri** | CAGCACCATTTTCCACTATTCCTCTT | 333-358 |  |  |

a Nucleotides numbering from the gene *cox1*.

b Temperature for the annealing/extension step.
